# Randomized phase III trial of a neoadjuvant regimen of four cycles of adriamycin plus cyclophosphamide followed by four cycles of docetaxel (AC4-D4) versus a shorter treatment of three cycles of FEC followed by three cycles of docetaxel (FEC3-D3) in node-positive breast cancer (Neo-shorter; NCT02001506)

**DOI:** 10.1007/s10549-023-06971-7

**Published:** 2023-06-26

**Authors:** Inhwan Hwang, Jeong Eun Kim, Jae Ho Jeong, Jin-Hee Ahn, Kyung Hae Jung, Byung Ho Son, Hak Hee Kim, Junyoung Shin, Hee Jin Lee, Gyungyub Gong, Sung-Bae Kim

**Affiliations:** 1 Department of Oncology, Asan Medical Center, University of Ulsan College of Medicine, 88 Olympic-ro 43-gil, Songpa-gu, Seoul, 05505 Republic of Korea; 2 Department of Surgery, Asan Medical Center, University of Ulsan College of Medicine, Seoul, Republic of Korea; 3 Department of Pathology, Asan Medical Center, University of Ulsan College of Medicine, Seoul, Republic of Korea; 4 Department of Radiology, Asan Medical Center, University of Ulsan College of Medicine, Seoul, Republic of Korea; 5 Department of Oncology, Daejeon Eulji Medical Center, Eulji University School of Medicine, Daejeon, Republic of Korea

**Keywords:** Neoadjuvant, AC followed by docetaxel, FEC followed by docetaxel, Operable breast cancer, Locally advanced breast cancer

## Abstract

**Purpose:**

To determine whether six cycles of FEC3-D3 has a comparable efficacy to eight of AC4-D4.

**Methods:**

The enrolled patients (pts) were clinically diagnosed with stage II or III breast cancer. The primary endpoint was a pathologic complete response (pCR), and the secondary endpoints were 3 year disease-free survival (3Y DFS), toxicities, and health-related quality of life (HRQoL). We calculated that 252 pts were needed in each treatment group to enable the detection of non-inferiority (non-inferiority margin of 10%).

**Results:**

In terms of ITT analysis, 248 pts were finally enrolled. The 218 pts who completed the surgery were included in the current analysis. The baseline characteristics of these subjects were well balanced between the two arms. By ITT analysis, pCR was achieved in 15/121 (12.4%) pts in the FEC3-D3 arm and 18/126 (14.3%) in the AC4-D4 arm. With a median follow up of 64.1 months, the 3Y DFS was comparable between the two arms (75.8% in FEC3-D3 vs. 75.6% in AC4-D4). The most common adverse event (AE) was Grade 3/4 neutropenia, which arose in 27/126 (21.4%) AC4-D4 arm pts vs 23/121 (19.0%) FEC3-D3 arm cases. The primary HRQoL domains were similar between the two groups (FACT-B scores at baseline, *P* = 0.35; at the midpoint of NACT, *P* = 0.20; at the completion of NACT, *P* = 0.44).

**Conclusion:**

Six cycles of FEC3-D3 could be an alternative to eight of AC4-D4. Trial registration ClinicalTrials.gov NCT02001506. Registered December 5,2013**.** https://clinicaltrials.gov/ct2/show/NCT02001506

**Supplementary Information:**

The online version contains supplementary material available at 10.1007/s10549-023-06971-7.

## Introduction

The previous national surgical adjuvant breast and bowel project (NSABP) B18 study [1] demonstrated no significant difference in overall survival (OS) or disease free survival (DFS) between adjuvant and neoadjuvant chemotherapy, also confirmed in many subsequent studies [2–4]. Although there has been no reported survival gain with neoadjuvant chemotherapy, it has been used to reduce the extent of local therapy or reduce delays in initiating therapy [1, 5–7]. In addition, some studies have confirmed that achieving a pathologic complete response (pCR) after neoadjuvant chemotherapy is significantly helpful in predicting long term survival outcomes [1–4]. Neoadjuvant chemotherapy has thus become a standard of care that can be considered for locally advanced breast cancer.

The previous randomized NSABP-B27 study reported a 90% overall clinical response rate after four cycles of AC followed by four cycles of docetaxel [8]. Three cycles of FEC (fluorouracil, epirubicin, and cyclophosphamide) followed by three cycles of docetaxel, compared to six cycles of FEC, in an adjuvant setting have also demonstrated a survival benefit [9]. Three cycles of FEC followed by three cycles of docetaxel (FEC3-D3) was a popular neoadjuvant chemotherapy regimen in europe when this study was designed. Six rather than eight cycles have an advantage in terms of a shorter treatment duration with lower toxicities and a higher compliance unless efficacy is compromised. Docetaxel can also be used at a dose of 75 mg/m^2^ in each cycle considering that the higher 100 mg/m^2^ dose showed no clinical benefit from the higher toxicity in previous studies [10, 11], and would be more feasible in a neoadjuvant setting in terms of a reduced toxicity and improved tolerance. However, there have been limited reports to date on whether efficacy is maintained, or quality of life (QoL) is reduced, when the number of treatment cycles is reduced. In our present study, we compared the degree of efficacy and QoL over the course of the neoadjuvant chemotherapy intervention in patients who underwent AC4-D4 or FEC3-D3 as a preoperative chemotherapy regiment for stage II or III breast cancer.

### Patients and methods

#### Study design and objectives

**Fig. 1:**
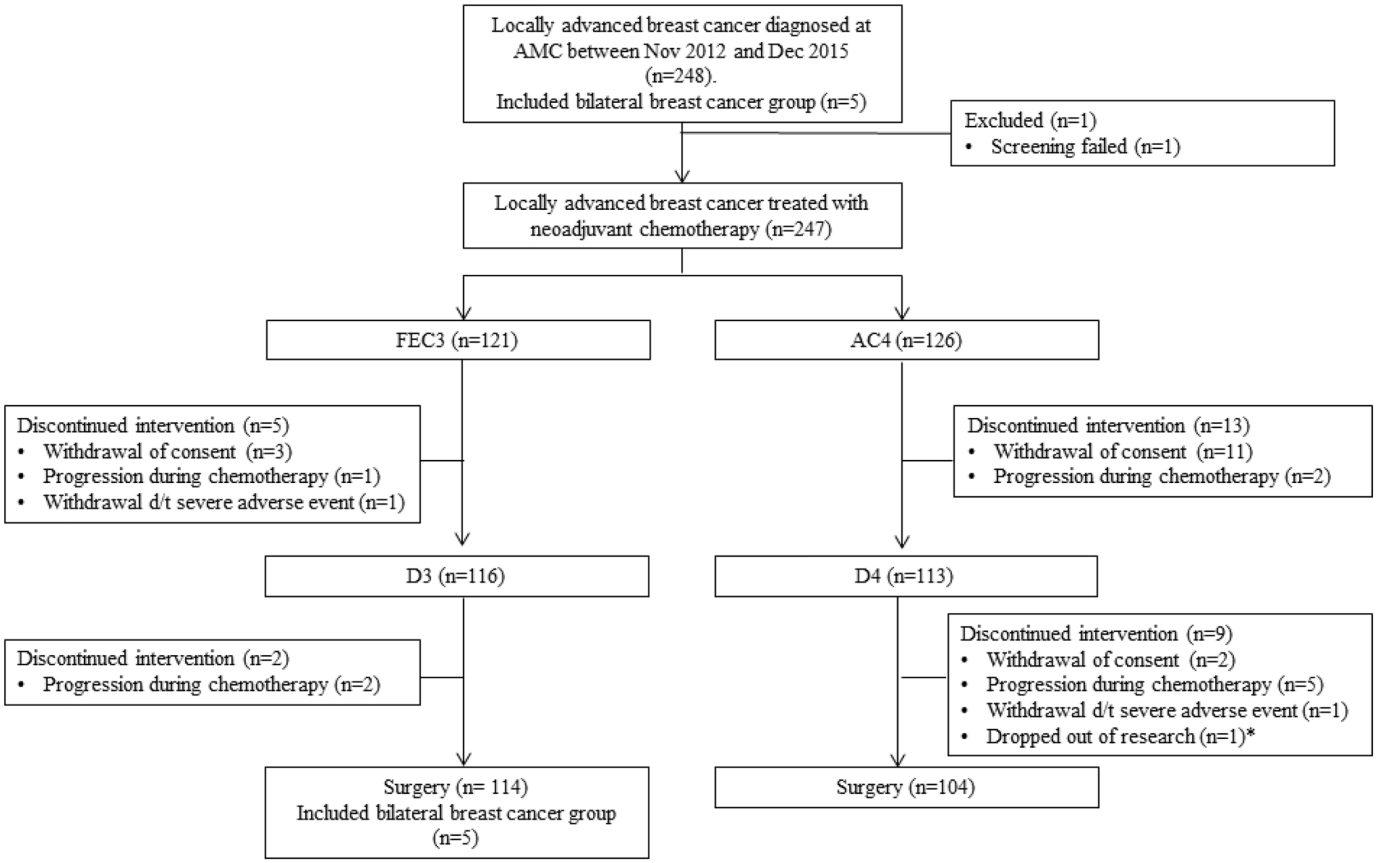
Study flow chart. *AC4* adriamycin, and cyclophosphamide (4 cycles), *AMC* Asan Medical Center, *D3* docetaxel (3 cycles), *D4* Docetaxel (4 cycles), *FEC3* fluorouracil, epirubicin, and cyclophosphamide (3 cycles). ^a^Dropped out because of an excessive rest period needed for recovery

This was a randomized, prospective, parallel group, comparative phase 3 study conducted at Asan Medical Center, Seoul, Korea. The patient allocation is outlined in Fig. 1. The primary outcome was pCR from a node-positive breast cancer treated with an FEC3-D3 or AC4-D4 neo-adjuvant chemotherapy regimen. Secondary outcomes included 3-year disease free survival (3Y DFS), quality of life (QoL), and the correlation between Ki-67 expression and pCR, which was defined as no evidence of invasive cancer in the breast or lymph nodes. Detailed descriptions of the study methodology and eligibility criteria are provided in the Supplementary Information.

#### Procedures

Three cycles of FEC followed by three cycles of docetaxel (FEC3-D3) were administered by intravenous injection every 3 weeks using the following dosages: 5-fluorouracil, 500 mg/m^2^; epirubicin, 100 mg/m^2^; cyclophosphamide, 500 mg/m^2^; and docetaxel, 75 mg/m^2^. Four cycles of AC followed by 4 cycles of docetaxel (AC4-D4) were also administered by intravenous injection every 3 weeks as follows: adriamycin, 60 mg/m^2^; cyclophosphamide, 600 mg/m^2^; and docetaxel, 75 mg/m^2^. Mammography and breast ultrasounds were done at the midpoint (after the three cycles of FEC in arm A and four cycles of AC in arm B) and at the completion of the chemotherapy. Breast magnetic resonance (MR) was performed at baseline and before surgery. Surgery was undertaken within 6 weeks of the last round of chemotherapy. The administration of adjuvant chemotherapy, hormonal therapy and/or trastuzumab, and postoperative radiation was at the discretion of the treating physician. The relative dose intensity (RDI) is the ratio of the actual dose intensity of chemotherapy delivered to the standard recommended dose intensity [12].

#### Response and toxicity assessments

Response assessments were done using RECIST version 1.1. Adverse events (AEs) were evaluated every 3 weeks (±1 week) using Common Terminology Criteria for Adverse Events (CTCAE) version 4.0. Patients who had received at least one cycle of chemotherapy were included in toxicity assessment. A QoL assessment was conducted at the midpoint and at the completion of the chemotherapy using Functional Assessment of Cancer Therapy-Breast (FACT-B) version 4.0 [13].

#### Follow up methods after the surgery

Post-op follow ups were done every 3 to 6 months for the first 2 years and then every 6 months for up to 5 years and included a physical examination, CBC, laboratory chemistry tests, and an annual mammogram with or without breast sonography. After then, follow ups were done annually.

#### Statistical analysis

With a two-sided type Ι error of 0.05 and a power of 80%, we calculated that 252 pts were needed in each treatment group to enable the detection of non-inferiority of neoadjuvant FEC3-D3 compared to AC4-D4 with a pCR rate of 20% (non-inferiority margin of 10%). Considering a dropout rate of 10%, and 280 pts in each arm, a total of 560 patients per arm would be enrolled. Pts were randomized using the stratified block randomization method with the hormone receptor and HER2 expression status included as the stratification factors. The sample size was amended due to slower enrolment and competing trials. The revised statistical procedure was that all parameters would be analyzed using descriptive statistics. Disease free survival was calculated with the Kaplan–Meier method. Categorical variables were expressed as proportions and continuous variables as the mean ± SD. The Mann–Whitney U-test was used to compare differences between the treatment arms. The Friedman test was used to detect repeated measurement difference. Statistical analysis was done using SPSS version 23.0 (IBM Corp, Armonk, NY), and statistical significance was defined as a *P* value less than 0.05. The cut off value of the Ki-67 labeling index was determined by the AUC curve based on the values of the highest sensitivity and specificity. The intention to treat (ITT) population was defined as all the patients who were randomized, excluding those who failed the screening. The per-protocol population was defined as patients who completed the study.

## Results

### Baseline characteristics of the total cohort

In this present study series, 248 patients diagnosed with stage II or III breast cancer between November 2012 and December 2015 were enrolled. These cases were randomly assigned (1:1) to an FEC3-D3 (n = 121, 48.9%) or AC4-D4 (n = 126, 51.1%) treatment arm. Subsequent to this enrollment, one patient was found to be ineligible for screening; 10 discontinued treatment due to progressive disease (7 in the AC4-D4 arm and 3 in the FEC3-D3 arm), 16 patients withdrew consent to participate (13 in the AC4-D4 arm and 3 in the FEC3-D3 arm), and three patients were unable to complete the study (2 in the AC4-D4 arm due to exceeding the dose delay limit of 9 weeks and grade 3 peripheral neuropathy, and one patient in the FEC3-D3 arm due to a loss of consciousness of unknown etiology). Ten out of the 247 patients (4.0%) experienced progression during the neoadjuvant chemotherapy. Two of them were unable to undergo surgery because they had a distant metastasis. The 218 remaining patients receiving surgery were included in our per-protocol analysis. The baseline characteristics were well balanced in terms of median age (49 vs 47), percentage of luminal type cases (66.1% vs 69.1%), and percentage of triple negative breast cancers (20.7% vs. 19.0%) between the FEC3-D3 (n = 121) and AC4-D4 (n = 126) arms. Clinical T2 (57.5% vs. 62.6%) and N1 (63.3% vs. 64.3%) stage tumors were also predominant in both arms (Table 1).

**Table 1:** Baseline characteristics of the study population

	Total (n = 247)	FEC3-D3 (n = 121)	AC4-D4 (N = 126)	P-value
Median age, y (range)	49 (29–74)	49 (29–68)	47 (31–74)	
≥ 65 y old, no (%)	8 (3.2%)	3 (2.5%)	5 (4.0%)	0.52
Menstrual state (%)				
Premenopausal	86 (35.0%)	37 (30.8%)	49 (38.9%)	0.19
Postmenopausal	161 (65.0%)	84 (69.2%)	77 (61.1%)	
TNM status				
T status (%)				
T1	38 (15.4%)	17 (14.2%)	21 (16.7%)	0.57
T2	149 (60.2%)	70 (57.5%)	79 (62.6%)	
T3	51 (20.7%)	29 (24.2%)	22 (17.5%)	
T4	9 (3.7%)	5 (4.1%)	4 (3.2%)	
N status (%)				
N0	4 (1.6%)	3 (2.5%)	1 (0.8%)	0.77
N1	158 (63.8%)	77 (63.3%)	81 (64.3%)	
N2	24 (9.8%)	12 (10.0%)	12 (9.5%)	
N3	61 (24.8%)	29 (24.2%)	32 (25.4%)	
Stage of disease(AJCC 7th)				
I	3 (1.2%)	3 (2.5%)	0 (0.0%)	0.31
IIA	19 (7.7%)	6 (4.9%)	13 (10.3%)	
IIB	100 (40.5%)	49 (40.5%)	51 (40.5%)	
IIIA	46 (18.6%)	25 (20.7%)	21 (16.7%)	
IIIB	18 (7.3%)	9 (7.4%)	9 (7.1%)	
IIIC	61 (24.7%)	29 (24.0%)	32 (25.4%)	
Subtype				
Luminal A	20 (8.1%)	9 (7.4%)	11 (8.8%)	0.87
Luminal B-like^a^	147 (59.5%)	71 (58.7%)	76 (60.3%)	
Triple negative^b^	49 (19.8%)	25 (20.7%)	24 (19.0%)	
HER2-positive^c^	31 (12.6%)	16 (13.2%)	15 (11.9%)	
Ki-67 labeling index				
Baseline ≥ 20%	185 (75.2%)	95 (79.2%)	90 (71.4%)	0.16
Baseline ≥ 55%	116 (47.2%)	60 (50.0%)	56 (44.4%)	0.38
Dec after NACT	168 (69.7%)	87 (72.5%)	81 (66.9%)	0.43

(1) Three patients with stage I disease were included in the study because the stage of the contralateral breast was locally advanced

(2) Four patients with N0 stage disease were included in this study because the stage of the contralateral breast was locally advanced in 3 out of 4 patients and the others had T2 lesions

*AC4-D4* adriamycin and cyclophosphamide (4 cycles) followed by docetaxel (4 cycles), *Dec* decreasing, *FEC3-D3* fluorouracil, epirubicin, and cyclophosphamide (3 cycles) followed by docetaxel (3 cycles), *HER2* human epidermal growth factor receptor 2, *NACT* neoadjuvant chemotherapy

^a^Luminal B-like: ER-positive (Allred score ≥ 3) with high Ki-67 (≥ 15%) or ER- with HER2-positivity

^b^triple negative: hormone receptor-negative [ER (Allred score < 3) and PR (Allred score < 3)] with HER2 negative (Her2/neu immunohistochemistry (IHC) < 1 + or Her2/neu 2 + and fluorescence in situ hybridization (FISH)-negative)

^c^HER2-positive: hormone receptor-negative with HER2 positivity (Her2/neu immunohistochemistry (IHC) 3 + or Her2/neu 2 + and fluorescence in situ hybridization (FISH)-positive)

### Pathologic complete response outcomes and correlations with the baseline Ki-67 labeling index

By intention-to-treat (ITT) analysis, pCR was achieved in 15/121 (12.4%) patients in the FEC3-D3 arm and 18/126 (14.3%) patients in the AC4-D4 arm. In the FEC3-D3 arm, 92/114 patients achieved a clinical response [4 complete responses (CR) and 88 partial responses (PR)] and among these cases, 15 patients (12.4%) achieved pCR. In the AC4-D4 arm, 95/104 patients achieved a clinical response (6 CR and 89 PR), among which 18 patients (14.3%) achieved pCR (Table 2). In terms of pCR, eight cycles were numerically slightly higher than six cycles even when analyzed by subtype (Table S2 in the Supplementary material). When different cut-offs for Ki 67 were assessed in the luminal B subtype, a Ki 67≥55% was associated with a higher pCR rate.

**Table 2:** Treatment efficacies determined by (A) intention to treat analysis and (B) per-protocol analysis

(A)		
	FEC3-D3^*^ (n = 121)	AC4-D4^**^ (n = 126)
Rate of pCR (n,%)	15 (12.4%)	18 (14.3%)
Three-year disease-free survival (DFS,%)	75.8%	75.6%
Hazard ratio (95% CI)	1.04 (0.64–1.70)	
Median 3-year DFS (months)	Not reached	Not reached
Clinical response		
Complete response	4 (3.3%)	6 (4.8%)
Partial response	88 (72.7%)	89 (70.6%)
Stable disease	22 (18.2%)	9 (7.1%)
Not evaluable	7 (5.8%)	22 (17.5%)
Type of surgery		
MRM	60 (50.0%)	64 (50.8%)
BCO	55 (45.8%)	50 (39.7%)
No surgery	6 (4.2%)	12 (9.5%)

(B)		
	FEC3-D3^*^ (n = 114)	AC4-D4^**^ (n = 104)
Rate of pCR (n,%)	15 (13.2%)	18 (17.3%)
Three-year disease-free survival (DFS,%)	77.0%	74.9%
Hazard ratio (95% CI)	1.06 (0.64–1.76)	
Median 3-year DFS (months)	Not reached	Not reached
Clinical response		
Complete response	4 (3.5%)	6 (5.8%)
Partial response	88 (77.2%)	89 (85.6%)
Stable disease	22 (19.3%)	9 (8.7%)
Type of surgery		
MRM	59 (51.8%)	57 (54.8%)
BCO	55 (48.2%)	47 (45.2%)

*AC4-D4* adriamycin and cyclophosphamide (4 cycles) followed by docetaxel (4 cycles), *BCO* breast conserving operation, *CI* confidence interval, *FEC3-D3* fluorouracil, epirubicin, and cyclophosphamide (3 cycles) followed by docetaxel (3 cycles), *MRM* modified radical mastectomy, *pCR* pathologic complete response

### Three-year disease-free survival outcomes

**Fig. 2:**
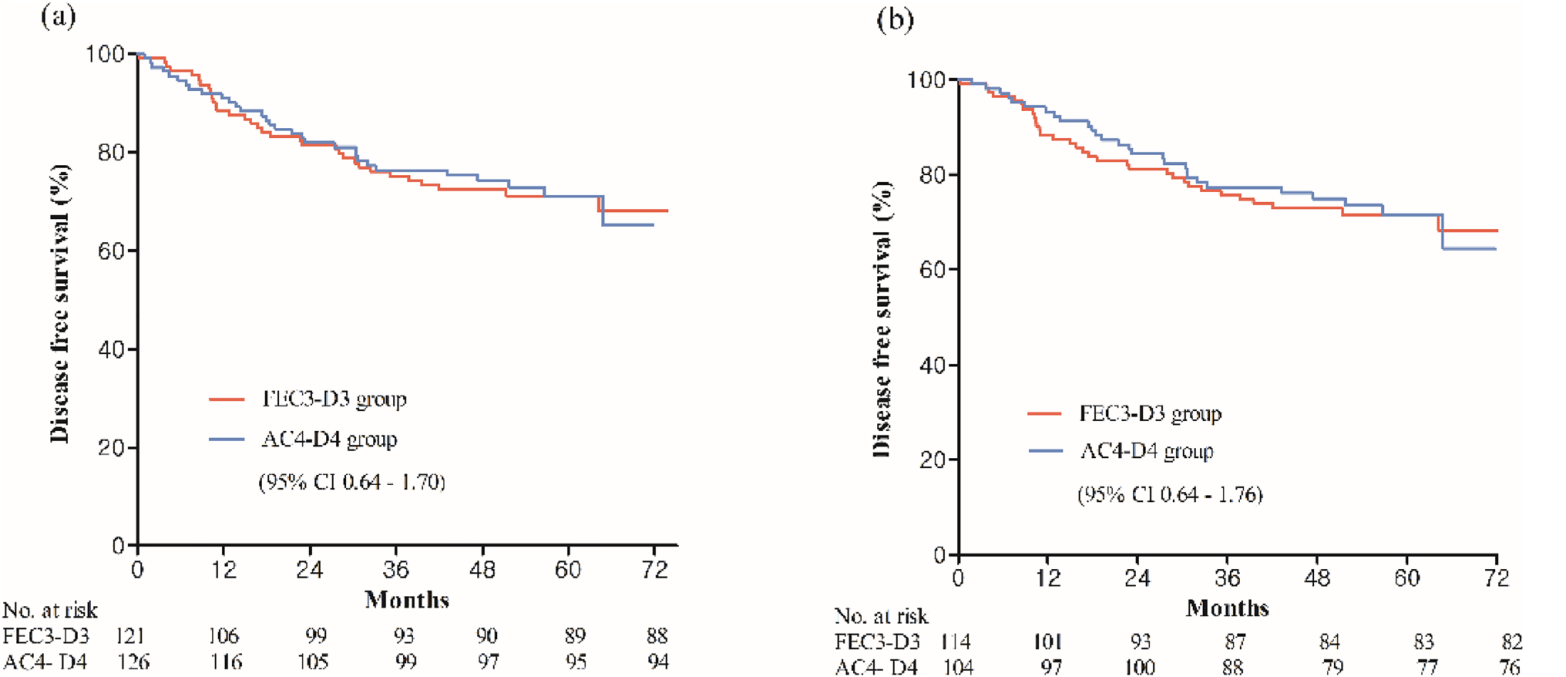
Kaplan–Meier plots of disease free survival outcomes. (**a**) Intention to treat. (**b**) Per protocol. *AC4-D4* adriamycin, and cyclophosphamide (4 cycles) followed by docetaxel (4 cycles), *CI* confidence interval, *FEC3-D3* fluorouracil, epirubicin, and cyclophosphamide (3 cycles) followed by docetaxel (3 cycles)

**Fig. 3:**
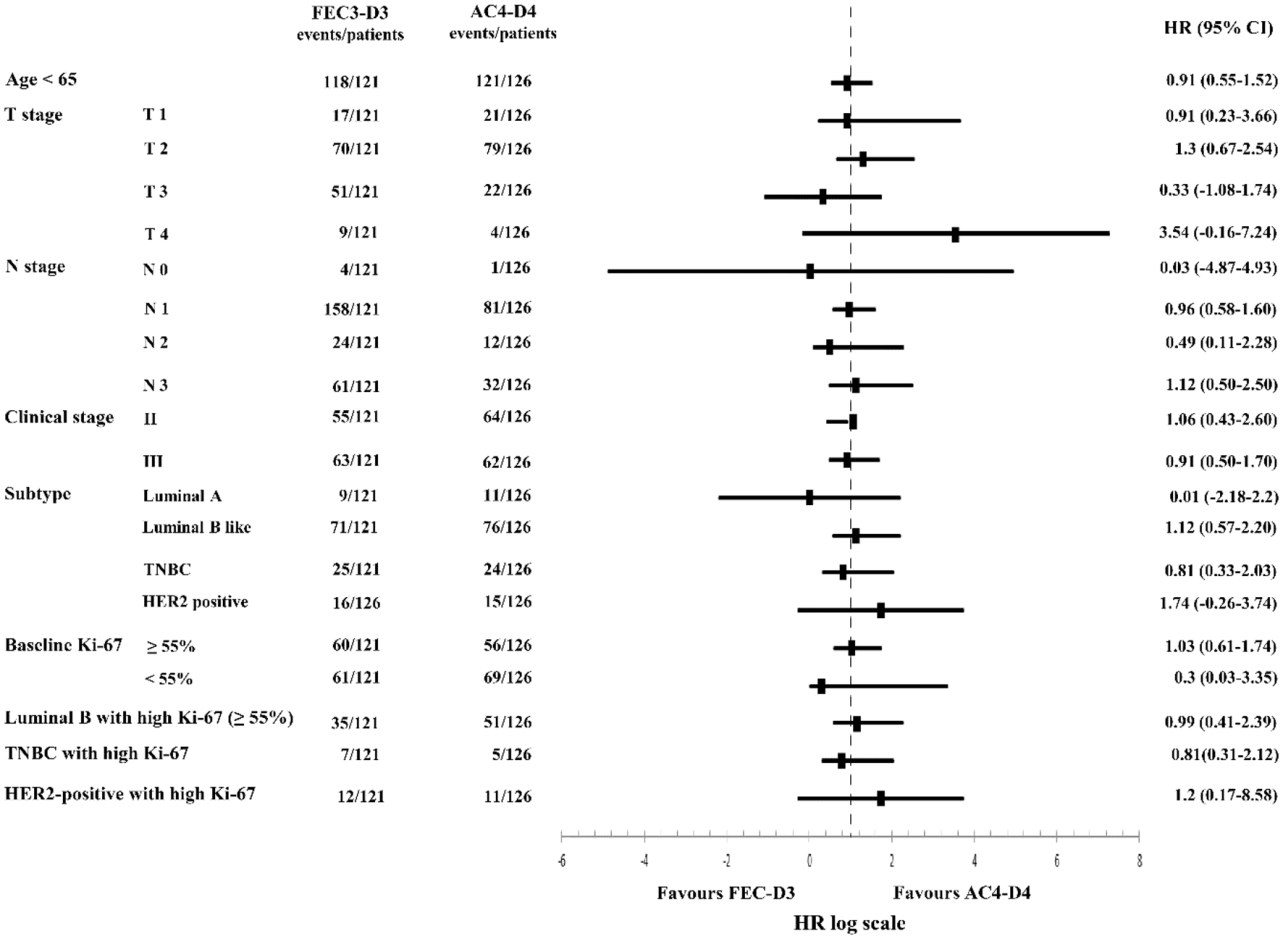
Subgroup analysis of 3 year disease free survival (3Y DFS) outcomes in the intention to treat population. *AC4-D4* adriamycin, and cyclophosphamide (4 cycles) followed by docetaxel (4 cycles), *CI* confidence interval, *FEC3-D3* fluorouracil, epirubicin, and cyclophosphamide (3 cycles) followed by docetaxel (3 cycles), *HER2* human epidermal growth factor receptor 2, *HR* hazard ratio, *TNBC* *triple negative breast cancer*

With a median follow up of 64.1 months, the 3Y DFS (75.8% in FEC3-D3 vs. 75.6% in AC4-D4) was comparable between the two arms Fig. 2A. Forest plots of the 3Y DFS for the subgroups in the ITT analysis are shown in Fig. 3. In the subgroup analysis, there was no favorable regimen between FEC3-D3 and AC4-D4. Univariate and multivariate analyses of the associations between the clinicopathologic factors and 3Y DFS are summarized in Table S1 in the Supplementary material. For the 3Y DFS, ≥ 55% of the baseline Ki-67 labeling index with luminal type (HR 2.1, 95% CI, 1.04–4.25), and ≥ 4 lymph node metastases at surgery (HR 1.9, 95% CI, 1.07–3.51) seemed to correlate with the 3Y DFS.

### Toxicity and QoL assessments

The most common AE was a Grade 3/4 neutropenia [27/126 (21.4%) patients in the AC4-D4 arm vs. 23/121 (19.0%) patients in the FEC3-D3 arm]. The most common Grade 3/4 non-hematologic AE was hyperglycemia (4.0%). A dose modification was made in 25/121 (20.7%) patients in the FEC3-D3 arm and 37/126 (29.4%) in the AC4-D4 arm (Table 3). The number of patients who completed chemotherapy were 114 out of 121 in the FEC3-D3 arm and 104 out of 126 in the AC4-D4 arm. A 20% dose modification was performed on 22 of 114 patients in the FEC3 group comprising three patients from cycle 1 and 19 patients from cycle 2. The relative dose intensity (RDI) for three cycles of FEC was 95.5%. Dose modification was performed in 6 of 114 patients in the D3 group. The RDI for three cycles of docetaxel was 99.2%. Dose modification was performed for 16 of 104 patients in the AC4 group. The RDI for 4 cycles of AC was 97.0%. A 20% dose modification was performed for 26 of 104 patients who had completed chemotherapy in the D4 group. The RDI for four cycles of docetaxel was 97.1%.

**Table 3:** Adverse events during neoadjuvant chemotherapy

	FEC3-D3 (n = 121)		AC4-D4^*^ (n = 126)	
	Grade 1–2	Grade 3–4	Grade 1–2	Grade 3–4
Hematologic events				
Neutropenia	4 (11.6%)	23 (19.0%)	11 (8.7%)	27 (21.4%)
FN		16 (13.2%)		17 (13.5%)
Anemia	12 (10.1%)	4 (0.8%)	8 (7.8%)	0 (0.0%)
Non-hematologic events				
Myalgia	78 (64.5%)	0 (0.0%)	25 (19.8%)	0 (0.0%)
Nausea	74 (61.2%)	0 (0.0%)	84 (66.7%)	0 (0.0%)
Constipation	33 (27.3%)	0 (0.0%)	42 (33.3%)	0 (0.0%)
Elevated ALT	21 (17.4%)	2 (1.7%)	15 (11.9%)	1 (0.8%)
Elevated AST	14 (11.6%)	1 (0.8%)	13 (10.3%)	0 (0.0%)
Anorexia	20 (16.5%)	0 (0.0%)	29 (23.0%)	0 (0.0%)
Edema	19 (15.7%)	0 (0.0%)	33 (26.2%)	0 (0.0%)
Skin rash	19 (15.7%)	0 (0.0%)	24 (19.1%)	0 (0.0%)
Insomnia	14 (11.6%)	0 (0.0%)	24 (19.1%)	1 (0.8%)
Hyperglycemia	12 (9.9%)	2 (1.7%)	12 (9.5%)	5 (4.0%)
Diarrhea	12 (9.9%)	0 (0.0%)	17 (13.5%)	0 (0.0%)
Mucositis	0 (0.0%)	0 (0.0%)	12 (9.5%)	1 (0.8%)
Neuropathy	0 (0.0%)	0 (0.0%)	7 (5.6%)	1 (0.8%)
Dose modification	25 (20.7%)		37 (29.4%)	

*AC4-D4* adriamycin and cyclophosphamide (4 cycles) followed by docetaxel (4 cycles), *ALT* alanine aminotransferase, *AST* aspartate aminotransferase, *FEC3-D3* fluorouracil, epirubicin, and cyclophosphamide (3 cycles) followed by docetaxel (3 cycles), *FN* febrile neutropenia

The QoL scores determined by FACT-B version 4 are listed in Table 4. The mean QoL values at baseline were 102.39, (standard deviation (SD), 17.50) vs.100.74 (SD, 16.72), at the midpoint of neoadjuvant chemotherapy were 85.24 (SD, 36.80) vs. 79.28 (SD, 38.62), and at the completion of chemotherapy were 75.71 (SD, 39.53) vs.70.73 (SD, 42.25) in the FEC3-D3 vs. AC4-D4 arms, respectively. In the FACT-B subgroups, emotional wellbeing (EWB) showed the lowest scores in both groups at baseline [FEC3-D3, 16.71 (SD, 4.81) vs. AC4-D4, 15.89 (SD, 4.79)]. Social wellbeing (SWB) had the lowest score in the FEC3-D3 arm [15.23 (SD,8.16)], whereas functional wellbeing (FWB) displayed the lowest score in the AC4-D4 arm [13.81 (SD, 7.61)], at the midpoint of the neoadjuvant chemotherapy. FWB was the lowest in both groups at the completion of chemotherapy [FEC3-D3, 12.83 (SD, 7.76) vs. AC4-D4, 12.72 (SD, 8.52)].

**Table 4:** Quality of life scores determined using Functional Assessment of Cancer Therapy-B (FACT-B) version 4.0

	Baseline			Mid-point of NACT			Completion of chemotherapy			
	FEC3-D3 median (SD) (min–max)	AC4-D4 median (SD) (min–max)	U-test^**a**^ *P*-value	FEC3-D3 median (SD) (min–max)	AC4-D4 median (SD) (min–max)	U-test^**a**^ *P*-value	FEC3-D3 median (SD) (min–max)	AC4-D4 median (SD) (min–max)	U-test^**a**^ *P*-value	Friedman test^**b**^ *P*-value
FACT-B (Range:0–148)	102.39(17.50) (50–146)	100.74 (16.72) (59–140)	0.35	85.24 (36.80) (0–130)	79.28 (38.62) (0–132)	0.20	75.71 (39.53) (0–135)	70.73 (42.25) (0–138)	0.44	< 0.001
PWB (Range: 0–28)	24.62 (3.64) (8–28)	24.87 (3.58) (10–28)	0.42	18.04 (8.31) (0–28)	17.63 (8.72) (0–28)	0.87	15.85 (8.88) (0–28)	14.58 (9.61) (0–28)	0.35	< 0.001
SWB (Range: 0–28)	18.12 (6.32) (0–28)	17.86 (6.71) (0–28)	0.88	15.23 (8.16) (0–28)	14.38 (7.60) (0–28)	0.29	13.52 (8.02) (0–27)	13.27 (8.49) (0–28)	0.89	< 0.001
EWB (Range: 0–24)	16.71 (4.81) (2–24)	15.89 (4.79) (0–24)	0.16	15.79 (7.20) (0–24)	14.63 (7.17) (0–24)	0.08	14.51 (7.72) (0–24)	12.85 (8.00) (0–24)	0.07	0.004
FWB (Range: 0–28)	17.57 (5.73) (2–28)	17.41 (5.91) (0–28)	0.76	17.57 (5.73) (2–28)	13.81 (7.61) (0–28)	0.31	12.83 (7.76) (0–28)	12.72 (8.52) (0–28)	0.99	< 0.001
BCS (Range:0–40)	25.38 (5.67) (9–38)	24.71 (6.17) (8–40)	0.488	21.44 (9.58) (0–35)	20.25 (9.87) (0–37)	0.30	19.00 (10.33) (0–37)	17.31 (10.66) (0–37)	0.13	< 0.001

*AC4-D4* adriamycin and cyclophosphamide (4 cycles) followed by docetaxel (4 cycles), *BCS* breast cancer subscale, *EWB* emotional wellbeing, *FEC3-D3* fluorouracil, epirubicin, and cyclophosphamide (3 cycles) followed by docetaxel (3 cycles), *FACT-B* functional Assessment of Cancer Therapy-B, *FWB* functional well-being, *NACT* neoadjuvant chemotherapy, *PWB* physical well-being, *SD* standard deviation, *SWB* social well-being

^a^U-test: the Mann–Whitney U-test was used to compare differences between the two treatment groups

^b^Friedman test: used to confirm whether a significant decrease in the QoL values occurred during the treatment period

## Discussion

FEC 3 followed by docetaxel 3 had been one of representative preoperative/adjuvant chemotherapy regimens specified in the NCCN guidelines up to 2017.[14-18]. Notably however, the FEC regimen was excluded from the NCCN guidelines after the NSABP-B36 trial [19]. In that study, six cycles of an FEC regimen did not show a superior efficacy to 4 AC cycles but did show a higher toxicity. Since the NSABP-B27 report, the AC4-D4 regimen has become widely used. However, the eight cycles of treatment in this protocol requires 6 months to complete, and there have been concerns regarding the reduction in patient compliance that is commonly related to a longer treatment duration. In addition, there is a reported QoL decrease due to increased exposure to anthracycline and taxane in the AC4-D4 regimen. The dose dense regimen has recently become widely used in the United states and Europe. There has also been a recent study demonstrating the superiority of the dose dense regimen [20]. However, at the beginning of our study in 2012, there was only a phase 2 study for dose-dense regimen and no randomized phase 3 trial. Also, in 2012, the dose dense regimen was not found to be superior by meta-analysis and was not available in daily clinical practice. Notably in this regard, the pCR and 3Y DFS showed no significant difference between our current study and two prior reports [21, 22], which investigated dose dense regimens as an NACT. Also, there was no significant difference in the 3Y DFS between a previous study[23] that used a dose dense regimen as post operative therapy and our current investigation. Prophylactic pegylated G-CSF (peg G-CSF) is required for a dose dense regimen and was not available as a primary prophylaxis at the beginning of this study.

The pCR rate in our present study series was low compared to the 26.1% level reported in the NSABP-B27 [8]. The pCR rate is known to be higher after neoadjuvant chemotherapy in the absence of HER2, estrogen receptor (ER) positivity and a lack of lymph node metastasis [24-27]. The different pCR rate between our current investigation and the NSABP B27 may have been due to the greater number of lymph node metastases [247/247 (100%) vs. 244/805 (30.3%)] and also the higher ER positivity [167/247 (67.6%) vs. 319/805 (39.6%) in Neo-Shorter vs. NSABP-B27]. In the NSABP-B27 study, the pCR rate in the AC followed by taxane treatment group with ER positivity was 14.1%, comparable to the 17.3% rate found in our present series. In addition, our observed pCR rate was low compared to that of a previous Indian study with a similar design concept [28]. The difference in the pCR rate between our present study and that prior Indian report may also have been due to differences in the proportion of triple negative breast cancers (TNBCs) and HER2-positive tumors, even though they have a similar clinical stage (Neo-Shorter vs. India, 32.4% vs. 49%). Also, in the prior study cohort from India, unlike our present series, HER2 2 + was considered to be negative without further HER2 in situ hybridization being conducted, which may have affected the findings.

Similar to previous studies, the higher Ki67 level among patients with the luminal type breast tumors in our present cohort was associated with a higher pCR. There was no significant correlation found between Ki-67 and the pCR rate in previous TNBC studies, or among these cases in our present study, and similar findings were also demonstrated in the prior Gepar TRIO trial [29]. However, there was a significant correlation found in our current analyses, in the luminal type, between the pCR and a Ki 67 index that was equal to or more than 55%.

Our current multivariate analysis revealed that a ≥ 55% baseline Ki-67 labeling index with luminal (HR 2.1, 95% CI,1.04–4.25), and ≥ 4 lymph node metastases at surgery (HR 1.9, 95% CI, 1.07–3.51) seemed to be correlated with the 3Y DFS outcome. A previous study found that an age > 50, higher T and N clinical stage, or tumor size > 5 cm were independent risk factors for distant metastasis in TNBC [30]. Additionally, in a previous meta-analysis study by Salvo et al. of hormone receptor-positive and HER2-negative breast cancers, it was confirmed that lymph-node positivity was an important factor for recurrence [31]. Our present results were consistent with the previously reported criteria for high-risk recurrence in TNBCs or hormone receptor-positive breast cancers. In terms of the 3Y DFS, the difference between our current study findings and those of the PACS01 trial appears be an effect of the inclusion ratio of stage I (neo-shorter: stage I, 0%, 3Y DFS, 77.0% vs. PACS01: stage I, 10.4%, 3Y DFS, 84.5%). Similarly, the 3Y DFS in the NSABP-B27 trial (5Y DFS, 71%) was comparable to that of the neo-shorter subjects treated with AC4-D4 (74.9%). The difference may be due to the presence of higher-risk patients in our current series, including those with more than 4 LN metastases [≥4LN metastases: Neo-Shorter vs. NSABP-B27, 85/247 (34.4%) vs. 114/752 (15.2%)].

In our current cohort, febrile neutropenia was within the 11–34% range reported in previous studies [9, 28]. A numerically larger number of patients withdrew their consent in AC4-D4 (n = 13 in AC4-D4, n = 3 in FEC3-D3). This withdrawal of consent was not necessarily related to the development of adverse events since there was a similar incidence of adverse effects between the two arms. Patients’ change of mind due to personal reasons unrelated to medical reasons was observed in four cases in AC4-D4 and two cases in FEC3-D3. Four patients in AC4-D4 requested to discontinue the chemotherapy due to individual intolerance rather than direct adverse effects, while there were none in FEC3-D3. A numerically high number of HER2-positive patients could not get trastuzumab added to docetaxel due to reimbursement issues (n = 5 in AC4-D4, n = 1 in FEC3-D3). Generally, myalgia is mainly known to be related to docetaxel. In this study, interestingly, myalgia was more common in the fewer cycles of docetaxel arm. Myalgia could occur by chance, but we assumed that the relatively lower dose modification in the FEC3-D3 group as compared with the AC4-D4 group (FEC-D3 vs. AC4-D4; 6/114 [5.2%] vs. 26/104 [25%]), which led to a relatively higher dose intensity (RDI) of docetaxel in the FEC3-D3 group compared with the AC4-D4 group (99.2% in the FEC3-D3 group vs. 97.1% in the AC4-D4 group).

In terms of QoL outcomes in our present cohort, FEC3-D3 was non inferior to AC4-D4 when FACT B scores at all points of time were compared. The FACT-B score, including all subfactors, showed a gradual decrease during chemotherapy. These differences indicated that the chemotherapy affected the QoL. At the treatment baseline, the EWB had the lowest score whereas the FWB score was the lowest at the completion of the chemotherapy. The lowest sub-factor before the start of the chemotherapy was the EWB, and this was likely related to the previously described prevalence of depression in breast cancer patients [32]. That prior study reported that upon a diagnosis of breast cancer, uncertainty about future disease progress, imagining of a poor situation by the patient, and fear of physical changes following treatment can cause depression. In our current investigation, it appeared that the EWB level before the start of chemotherapy was also influenced by the aforementioned factors. The lowest subfactor at the completion of chemotherapy was the FWB, likely because of the toxicity effects after these treatments. Interestingly, at the midpoint of treatment, the FEC3-D3 cases had the lowest SWB and AC4-D4 patients had the lowest FWB. The FWB was thus not the lowest in the FEC3-D3 group even in the middle of the chemotherapy. The cause of this might be associated with the decrease in anthracycline administration but further research is warranted.

## Limitations

There were some limitations of our current study of note. Although the results of the TRYPHAENA trial were published in 2013 [33], we were unable to use a HER2 blockade in our current neoadjuvant setting and study period because of the reimbursement policy of the Korean National Health Insurance system for locally advanced breast cancer. Hence, 12.6% (31/247) of the cases included in our present study series had the HER2 subtype. In addition, only the 3Y DFS outcomes could be confirmed among our study patients because of the relatively short follow-up period. In this regard, continuous follow-up will be required to confirm any differences in the long-term outcomes in both arms. Since the sample size was smaller than planned, we could not discriminate whether factors were insignificant due to this reduced sample size or were truly not meaningful. It may thus be necessary to conduct further research in larger cohorts. In this present study, there were fewer patients aged 65 or older (3.2%, 8/247). Considering that aging is a trend in Asian countries, it would be good to have additional studies to confirm our present findings in patients aged 65 or older.

## Conclusion

Six cycles of chemotherapy is a potentially viable option for patients who cannot tolerate 8 cycles due to age, time or co-morbidities.

## Supplementary Information

Below is the link to the electronic supplementary material.

Supplementary file1 (DOCX 49 KB)

## Data Availability

All data and materials will be made available upon reasonable request.

## Declarations

### Research involving human and animal participants

All of the enrolled human subjects in this study provided written informed consent to participate and to the publication of the findings.
