# Supplementary material for: Randomized phase III trial of a neoadjuvant regimen of four cycles of adriamycin plus cyclophosphamide followed by four cycles of docetaxel (AC4-D4) versus a shorter treatment of three cycles of FEC followed by three cycles of docetaxel (FEC3-D3) in node-positive breast cancer (Neo-shorter; NCT02001506)
Source: Breast Cancer Res Treat. 2023 Jun 26;201(2):193–204. doi: 10.1007/s10549-023-06971-7 (PMC10361883; doi:10.1007/s10549-023-06971-7)
Supplement: Supplementary file 1 — Supplementary file1 (DOCX 49 KB) [file 10549_2023_6971_MOESM1_ESM.docx]

**Randomized phase III trial of a neoadjuvant regimen of four cycles of adriamycin plus cyclophosphamide followed by four cycles of docetaxel (AC4-D4) versus a shorter treatment of three cycles of FEC followed by three cycles of docetaxel (FEC3-D3) in node-positive breast cancer (Neo-Shorter; NCT02001506)**

Inhwan Hwang, et al.

**Appendix/Supplemental Material**

**Supplementary Methods p. 2**

**Supplementary Table 1 p. 3**

**Supplementary Table 2 p. 4**

**Supplementary Methods**

**Randomization of the study subjects**

Patients were stratified according to their 1) hormonal receptor status [ER- or PR-positive (Allred score ≥ 3) vs. -negative (Allred score<3)]; and 2) HER2 expression status [HER2-positive:Her2/neu immunohistochemistry (IHC) 3+ or Her2/neu 2+ and fluorescence in situ hybridization (FISH)-positive vs. -negative: Her2/neu IHC 0-1 or Her2/neu 2+ and FISH negative]. These cases were then randomly assigned (1:1) to receive either arm A (FEC3-D3) or arm B (AC4-D4) treatments using the stratified block randomization method without blinding. The randomization table was generated by independent personnel who had no other involvement with this study, and the block sizes were 4 or 6.

**Eligibility criteria**

1. **Inclusion criteria**

Subjects met the following criteria to be eligible for participation in this study:

1. Aged 20 years or older with histologically proven breast cancer (including inflammatory breast
   cancer) of clinical stage II or III with a histologically proven lymph-node involvement.
2. An Eastern Cooperative Oncology Group (ECOG) performance status of less than 2.
3. Adequate hematological function (absolute neutrophil count ≥ 1.5 x 10^9^/L, platelet count ≥ 100 x 10^9^/L and hemoglobin level > 9 g/dL)
4. Adequate liver and kidney function [AST/ALT ≤ 1.5 x upper limit of normal (ULN), serum
   bilirubin < 1.5 ULN, and alkaline phosphatase < 1.5 x ULN, serum creatinine < 1.5 x ULN]
5. **Exclusion criteria**

Subjects meeting any of the following criteria were excluded from the study:

1. Pregnant or lactating
2. Documented history of cardiac disease contraindicating the use of anthracyclines (cardiac ejection
   fraction <50%),
3. Double primary cancer (except bilateral breast cancer)
4. Active uncontrolled infection.
5. A history of chemotherapy or radiotherapy interventions for any malignancy (exceptions were
   recognized basal cell carcinoma of skin, cervical carcinoma *in situ*, controlled differentiated
   thyroid cancer, where the patient has been disease-free for 10 years, and where the treatment
   had been solely a resection)

**Supplementary Table S1** Univariate and multivariate analyses for 3-year disease free survival using a Cox regression model

| Variables | Univariate analysis | | | Multivariate analysis | | |
| --- | --- | --- | --- | --- | --- | --- |
|  | HR | 95% CI |  | HR | 95% CI |  |
| Age, years (≥65) | 0.60 | 0.09-4.56 |  |  |  |  |
| CTx ((FEC3-D3 vs. AC4-D4) | 0.94 | 0.57-1.57 |  |  |  |  |
| Down stage after NACT | 1.28 | 0.76-2.13 |  |  |  |  |
| Baseline Ki-67 (≥20%) with luminal | 2.11 | 1.04-4.30 |  |  |  |  |
| Baseline Ki-67 (≥55%) with luminal | 1.78 | 0.94-3.37 |  | 2.1 | 1.04-4.25 |  |
| Subtype of breast cancer |  |  |  |  |  |  |
| Luminal A | 3.15 | 0.77-12.92 |  |  |  |  |
| Luminal B like | 1.31 | 0.79-2.19 |  |  |  |  |
| TNBC | 2.78 | 1.61-4.81 |  |  |  |  |
| HER2 positive | 1.60 | 0.64-4.03 |  |  |  |  |
| Post-surgery Ki67 (≥15%) with luminal | 2.41 | 1.44-4.04 |  |  |  |  |
| Ki67reduction after NACT | 1.50 | 0.87-2.56 |  |  |  |  |
| Ki67reduction after NACT (≥∆40%) | 1.87 | 1.13-3.12 |  |  |  |  |
| Ki67reduction after NACT (≥∆50%) | 1.77 | 1.07-3.00 |  |  |  |  |
| Post-surgery HG (1,2 vs 3) | 1.90 | 0.26-13.77 |  |  |  |  |
| Post-surgery NG (1,2 vs 3) | 1.55 | 0.21-11.21 |  |  |  |  |
| Post-surgery residual tumor (≥5.0cm) | 1.77 | 1.0-3.28 |  |  |  |  |
| Post-surgery residual LN (≥4) | 2.08 | 1.22-3.56 |  | 1.94 | 1.07-3.51 |  |

**Abbreviations: AC4-D4,** *adriamycin, and cyclophosphamide (4 cycles) followed by docetaxel (4 cycles)*; **CI,** *confidence interval;* **∆,** *Delta;* **CTx,** *chemotherapy;* **FEC3-D3,** *fluorouracil, epirubicin, and cyclophosphamide (3 cycles) followed by docetaxel (3 cycles)*; **HER2,** *human epidermal growth factor receptor 2***; HG,** *histologic grade*; **HR**, *Hazard ratio*; **LN,** *lymph node*; **NACT*,*** *neoadjuvant chemotherapy*; **NG,** *nuclear grade*

**Supplementary Table S2:** Summary of treatment efficacies determined from the per protocol analysis for each disease subtype.

1. Luminal type

|  | **FEC3-D3**^*^  **(n=78)** | | **AC4-D4**^**^  **(n=75)** | |  |
| --- | --- | --- | --- | --- | --- |
| Rate of pCR (n, %) | 4 (5.1%) | | 8 (10.6%) | |  |
| Three-year disease-free survival (DFS, %) | 81.5% | | 81.8% | |  |
| Hazard ratio (95% CI) | 0.96(0.50-1.85) 0.91 | | | | |
| Median 3-year DFS (months) | Not reached | Not reached | |  | |
| Clinical response  Complete response  Partial response  Stable disease | 2 (2.6%)  59 (75.6%)  17 (21.8%) | | 2 (2.7%)  66 (88.0%)  7 (9.3%) | |  |

1. TNBC

|  | **FEC3-D3**^*^  **(n=23)** | | **AC4-D4**^**^  **(n=18)** | |  |
| --- | --- | --- | --- | --- | --- |
| Rate of pCR (n, %) | 5 (21.7%) | | 7 (38.9%) | |  |
| Three-year disease-free survival (DFS, %) | 50.0% | | 55.6% | |  |
| Hazard ratio (95% CI) | 0.81(0.33-2.03) 0.66 | | | | |
| Median 3-year DFS (months) | Not reached | Not reached | |  | |
| Clinical response  Complete response  Partial response  Stable disease | 0 (0.0%)  20 (87.0%)  3 (13.0%) | | 4 (22.2%)  14 (77.8%)  0 (0.0%) | |  |

1. HER2-positive

|  | **FEC3-D3**^*^  **(n=13)** | | **AC4-D4**^**^  **(n=11)** | | ***P* value** |
| --- | --- | --- | --- | --- | --- |
| Rate of pCR (n, %) | 6 (46.2%) | | 3 (27.3%) | |  |
| Three-year disease-free survival (DFS, %) | 84.6% | | 81.8% | |  |
| Hazard ratio (95% CI) | 1.74(0.29-10.4) 0.91 | | | | |
| Median 3-year DFS (Months) | Not reached | Not reached | |  | |
| Clinical response  Complete response  Partial response  Stable disease | 2 (15.4%)  9 (69.2%)  2 (15.4%) | | 0 (0.0%)  9 (81.8%)  2 (18.2%) | |  |

**Abbreviations: AC4-D4,** *adriamycin and cyclophosphamide (4 cycles) followed by docetaxel (4 cycles)*; **CI,** *Confidence interval;* **FEC3-D3,***fluorouracil, epirubicin, and cyclophosphamide (3 cycles) followed by docetaxel (3 cycles)*; **pCR,** *pathologic complete response*; **TNBC,***triple negative breast cancer.*

`
